# Atypical Hemolytic Uremic Syndrome: A Review of Complement Dysregulation, Genetic Susceptibility and Multiorgan Involvement

**DOI:** 10.3390/jcm14072527

**Published:** 2025-04-07

**Authors:** Razvan-George Bogdan, Paula Anderco, Cristian Ichim, Anca-Maria Cimpean, Samuel Bogdan Todor, Mihai Glaja-Iliescu, Zorin Petrisor Crainiceanu, Mirela Livia Popa

**Affiliations:** 1 Plastic Surgery Department, “Victor Babes” University of Medicine and Pharmacy, 300041 Timisoara, Romania; razvan.bogdan@umft.ro (R.-G.B.); acimpeanu@umft.ro (A.-M.C.); mihai.iliescu.glaja@umft.ro (M.G.-I.); crainiceanu.zorin@umft.ro (Z.P.C.); 2 County Clinical Emergency Hospital Pius Branzeu, 300723 Timisoara, Romania; 3 Faculty of Medicine, “Lucian Blaga” University of Sibiu, 550024 Sibiu, Romania; samuelbogdant@gmail.com (S.B.T.); liviamirelapopa@yahoo.com (M.L.P.)

**Keywords:** atypical hemolytic uremic syndrome, thrombotic microangiopathy, complement, aHUS, C5 inhibitors, eculizumab, ravulizumab

## Abstract

Atypical hemolytic uremic syndrome (aHUS) is a rare, life-threatening thrombotic microangiopathy (TMA) characterized by complement dysregulation, leading to microvascular thrombosis and multi-organ injury. TMAs are defined by thrombocytopenia, microangiopathic hemolytic anemia and organ dysfunction caused by small-vessel thrombosis. Unlike thrombotic thrombocytopenic purpura, which results from severe ADAMTS13 deficiency, aHUS is driven by uncontrolled activation of the alternative complement pathway. While the kidneys are most frequently affected, other vital organs can also be involved. Genetic susceptibility contributes significantly to disease risk, but a trigger such as infection, pregnancy or autoimmune disease is usually required. Diagnosis is challenging due to overlapping features with other TMAs and relies on exclusion and complement testing. C5 inhibitors, such as eculizumab and ravulizumab, have revolutionized treatment but necessitate prophylactic vaccination and ongoing clinical surveillance. While these therapies provide effective disease control, discontinuing treatment remains complex, especially in patients with complement gene mutations. New therapies targeting various points in the complement cascade are under investigation and may offer safer, more cost-effective options. Progress in genetic profiling and biomarker discovery is essential for earlier diagnosis, individualized therapy and relapse prevention. This review highlights recent advances in the understanding of aHUS pathophysiology, clinical features and evolving therapeutic strategies aimed at improving patient outcomes.

## 1. Introduction

Thrombotic microangiopathy (TMA) is a histopathological classification that encompasses a spectrum of vascular thrombotic irregularities observed in various conditions, including hemolytic uremic syndrome (HUS), hemolysis and thrombotic thrombocytopenic purpura (TTP), low platelet counts and elevated liver enzymes [1]. Although these conditions have distinct etiologies, they exhibit similar pathological characteristics, including thrombotic vascular occlusion, which leads to ischemia and subsequent tissue injury [2]. Clinically, they are characterized by consumptive thrombocytopenia, microangiopathic hemolytic anemia (MAHA) and multi-organ dysfunction, with the kidneys being the most commonly affected, though other organs may also be involved [3,4,5,6].

Atypical hemolytic uremic syndrome (aHUS) represents a unique pathological entity within the broader spectrum of TMA, occurring equally in adults and children, with no significant gender differences [7,8,9]. It is marked by the presence of MAHA, a reduction in platelet count and blockage of small blood vessels, ultimately inducing injury to vital organs, such as the brain, ocular structures, heart, gastrointestinal tract and kidneys [10]. This disorder stems from an imbalance in the regulation of the alternative complement pathway and can be life-threatening if not promptly and properly managed [8,10]. Due to its clinical overlap with other forms of TMA, especially TTP and HUS, early and accurate diagnosis is essential to initiate appropriate treatment [11]. Delayed diagnosis is associated with increased risk of irreversible kidney injury, prolonged hospitalization and increased healthcare costs [12,13].

aHUS is an exceptionally rare pathological disorder, with an estimated prevalence ranging between 0.5 and 2 cases per million individuals [3,14]. Global epidemiological data suggest similar incidence rates across Europe, North America and Asia, though underreporting in low-resource settings likely leads to underestimation [15,16]. This condition can manifest across all stages of human development, from the neonatal phase to adulthood [17]. In pediatric cohorts, aHUS constitutes approximately 11% of all HUS cases [3,18]. The onset during childhood (≤18 years) is observed at a slightly lower frequency compared to adulthood, with pediatric cases accounting for approximately 40% and adult cases comprising around 60% of occurrences [10,19].

The typical clinical course begins with acute kidney injury, which can progress to end-stage renal disease (ESRD) in the absence of timely therapy [20]. During the acute phase, neurological, cardiovascular and gastrointestinal complications may also occur [21]. Long-term consequences include chronic kidney disease, hypertension, treatment dependency, reduced quality of life and psychological burden, particularly in pediatric patients [22,23,24].

Genetic mutations are detected in approximately 60% of aHUS cases, but disease onset typically requires an additional trigger [25]. While genetic alterations increase susceptibility, a precipitating event is necessary for clinical manifestation, making aHUS a “double-hit” disease [26,27,28].

TTP is primarily caused by a severe deficiency under 10% of the ADAMTS13 enzyme, which normally cleaves ultra-large von Willebrand factor multimers to prevent excessive platelet aggregation [2]. The functional interplay between ADAMTS13 and von Willebrand factor is crucial in preventing platelet-rich thrombus formation, distinguishing TTP pathophysiology from complement-mediated TMA. In contrast, aHUS is driven by dysregulation of the alternative complement pathway, leading to uncontrolled complement activation and endothelial injury [10]. TMA can also develop after solid organ or hematopoietic stem cell transplantation, triggered by immunosuppressive agents or ischemia–reperfusion injury [29].

The absence of a definitive diagnostic method complicates the identification of aHUS [7]. In recent years, research has shed light on the mechanisms and clinical features of aHUS, improving its classification and understanding. Advances in evidence have led to targeted therapies for certain types, significantly reducing complications and deaths. However, treatments for other variants remain general and reactive. Given the complex interplay of genetic and environmental triggers, early recognition and precise diagnosis are critical to guide therapy, avoid organ failure and reduce mortality [14].

This review aims to provide a comprehensive synthesis of current knowledge on aHUS pathophysiology, clinical manifestations, diagnostic challenges and therapeutic advances, while highlighting differences between pediatric and adult cases and discussing future directions for personalized care.

## 2. Genetic Factors and Disease Mechanisms

Comprehensive genomic analyses in aHUS have identified a spectrum of rare pathogenic variants in complement-associated genes, alongside large-scale genomic rearrangements [30]. Notably, the more stringent the exclusion criteria for secondary forms of HUS, the greater the observed prevalence of deleterious mutations in genes regulating complement activation [31].

Primary aHUS can be hereditary or acquired, resulting from genetic mutations or autoantibodies that disrupt complement regulation, leading to uncontrolled alternative pathway activation [26,32]. The complement system is an essential element of innate immunity, triggered via three distinct pathways: the classical, the lectin and alternative pathways [31]. In the alternative pathway, spontaneous hydrolysis of C3 leads to the formation of C5 convertase, which subsequently cleaves C5 into C5a, a potent chemoattractant and C5b, the initial component of the membrane attack complex (MAC) C5b-9 [28,33,34]. Dysregulated complement activity promotes abnormal MAC formation on endothelial cells, particularly in the renal vasculature, causing severe damage and serving as the primary pathogenic mechanism in aHUS [28,35].

A substantial number of these genetic alterations, predominantly identified within CFH, CFB, MCP, CFI and C3, encompass both loss-of-function and gain-of-function mutations [33,36]. These affect either key complement regulatory proteins (MCP, CFI and CFH) or essential components of the C3 convertase (CFB and C3), thereby disrupting homeostatic control of complement activity [34,36]. The CFH, CFI, C3 and CFB genes encode circulating plasma glycoproteins, primarily synthesized by hepatocytes, whereas MCP encodes CD46, a transmembrane regulatory protein expressed on various cellular surfaces [33,36].

Approximately 60–70% of patients with aHUS have identifiable genetic or acquired abnormalities in complement-regulating components, with the most frequently affected genes being CFH (20–30%), CFI (5–10%) and C3 (2–10%) [10,14]. In these cases, disease onset often requires a secondary trigger, such as infection, pregnancy or autoimmune disease, illustrating the “double-hit” model of pathogenesis [37]. For example, a carrier of a pathogenic CFI mutation may remain asymptomatic for years until exposed to a triggering event, such as upper respiratory infection, diarrhea or gastroenteritis [27].

The interplay between complement mutations and environmental triggers is now recognized as central to disease initiation and severity and is being actively explored through international cohorts, such as the Global aHUS Registry [15,19].

Penetrance varies by mutation type: CFH mutations have an estimated penetrance of 50%, while MCP mutations are associated with a lower penetrance of approx. 20% and better prognosis [38]. Table 1 summarizes the most relevant genes implicated in aHUS pathogenesis, highlighting their protein products, mutation effects, estimated prevalence among patients and known clinical correlations.

FHAAs are autoantibodies that disrupt factor H (FH), a key regulator of the complement system, leading to immune dysregulation in diseases like aHUS and C3G [43]. In aHUS, FHAAs primarily target the C-terminal SCR19-20 domains, impairing FH’s ability to protect endothelial cells from complement attack [31,44]. Similarly, CFH mutations, particularly clustered in SCR19-20, are the most common genetic abnormalities linked to aHUS, highlighting this region’s critical role in complement regulation [31,44].

A significant proportion of FHAAs in aHUS cases correlate with a homozygous deletion of CFHR1, whose SCR4-5 domain closely resembles CFH SCR19-20 [45,46]. This structural similarity may trigger cross-reactive autoantibody formation, though the exact molecular mechanism remains unclear. FHAAs are more common in children, affecting approximately 10–15% of pediatric aHUS cases, with prevalence reaching up to 50% in Indian populations [47,48]. In adults, they occur less frequently and are sometimes associated with monoclonal gammopathy [49].

Despite their clinical importance, the standardization of FHAA detection remains a challenge. Variability in assay techniques and cutoff thresholds may lead to false positives or underdiagnosis, particularly in early or subclinical disease [50]. While ELISA remains the most common method, it primarily measures free circulating autoantibodies and may overestimate their pathogenic relevance [51]. The presence of circulating FH–FHAA immune complexes may correlate more closely with disease activity, but these are not routinely measured in all centers, potentially delaying appropriate diagnosis and management.

Accurate detection of FHAAs is essential for diagnosis and treatment. While ELISA is widely used, it mainly identifies free FHAAs and may overestimate binding affinity [52,53]. Since FH-FHAA complexes correlate better with disease activity, improved detection methods are needed. A newly developed immunochromatographic test allows for the rapid visual detection of FHAAs (IgG and IgM) and quantification of FH-FHAA complexes directly from serum or plasma, potentially enhancing diagnostic speed and accessibility [54,55].

## 3. aHUS Manifestations

aHUS is a complex disorder that leads to diverse clinical manifestations. Renal impairment frequently manifests as proteinuria, hematuria, hypertension and azotemia, with proteinuria typically being mild but occasionally reaching nephrotic-range levels, while many patients ultimately require renal replacement therapy [32]. Renal involvement is nearly universal at presentation, with 60–70% of patients developing acute kidney injury and up to 50% progressing to end-stage renal disease within a few years if not treated promptly [25,56]. The severity of renal dysfunction at onset is a key prognostic indicator, with early response to complement inhibition associated with improved long-term kidney outcomes [21].

Beyond renal involvement, aHUS can impact multiple physiological systems, including the nervous, gastrointestinal, cardiovascular, integumentary, respiratory and ocular systems [21]. In many patients with complement risk factors, a triggering event, such as autoimmune disease, transplantation, pregnancy, infection, medication or metabolic disorder, is needed for aHUS to manifest [38,57]. While some extra-renal complications arise during the acute phase, others develop as long-term consequences of persistent complement activity [21]. These manifestations arise through systemic endothelial injury and widespread microvascular thrombosis mediated by dysregulated complement activation, particularly excessive C5 activation and MAC deposition [8,10,12,33].

The pathogenesis of aHUS is driven by microangiopathy and endothelial injury resulting from excessive C5 activation and MAC formation [10]. This endothelial damage triggers thrombus formation, platelet consumption and erythrocyte fragmentation, leading to TMA, which is characterized by impaired kidney function, low platelet count and destruction of red blood cells [21].

### 3.1. Cardiovascular and Pulmonary Involvement

Although the small blood vessels in the kidneys are mainly impacted, heart and blood vessel complications have also been observed, affecting both narrow and major arteries [58]. These include left ventricular hypertrophy, hypertrophic and dilated cardiomyopathy, increased CK-MB enzyme levels, improper valve function, intracardiac blood clots and an abnormally fast heartbeat [59]. Hypertension, often moderate to severe, results from a combination of vascular disease and volume expansion, further complicating disease management [10,32].

In pediatric patients, cardiovascular involvement has been reported in up to 43% of cases, while in adults, it ranges from 3–10% [59,60,61]. Although heart complications in aHUS can partly result from high blood pressure and excess fluid buildup due to sudden kidney failure, instances of heart muscle and blood vessel damage occurring independently of these factors suggest a direct injury to cardiac tissues driven by complement system activation [59,62]. The presence of cardiac involvement at presentation has been associated with increased risk of ICU admission, need for dialysis and worse renal outcomes [63].

Lung-related complications in aHUS usually arise as part of widespread organ dysfunction, frequently manifesting as pulmonary fluid accumulation due to heart dysfunction and/or excessive fluid retention [64]. Respiratory failure requiring mechanical ventilation occurs in up to 21% of pediatric patients, typically secondary to pulmonary edema [65]. In rarer instances, pulmonary embolism and hemorrhage have been documented in a small number of cases [66,67]. Pulmonary complications are thought to result from thrombotic microangiopathy in pulmonary capillaries or from indirect cardiac dysfunction [68].

### 3.2. Dermatologic and Systemic Signs

Cutaneous and circulatory complications have been documented in a limited number of published cases of aHUS [69]. Dermatological manifestations reported in the medical literature range from skin eruptions to distal tissue necrosis and gangrene [70]. When affecting young pediatric patients, cutaneous and vascular involvement tends to be severe and frequently emerges as an early systemic indicator of the condition [71,72]. Skin involvement may also occur in adult patients and may be an indicator of systemic complement activation, even in the absence of hematological criteria [69]. In some instances, dermatologic abnormalities may appear in the absence of anemia or low platelet counts, potentially signaling sustained complement system activation despite the absence of other biochemical markers commonly linked to aHUS [69].

### 3.3. Neurological and Ocular Manifestations

Neurologic complications rank among the most frequent aHUS extra-renal manifestations, with reported incidence ranging from 8% to 48% of cases [18,58]. Findings from the Turkish pediatric aHUS registry reveal central nervous system involvement in 27.2% of cases [58,60]. The clinical spectrum is broad, encompassing seizures, visual impairment, hemiparesis, headaches, altered consciousness, hallucinations and encephalopathy [65]. Additional neurological findings include cognitive disturbances, agitation, diminished reflexes, focal deficits, diplopia, nystagmus, hemiplegia and, in severe cases, coma [18,21]. Neurological involvement at onset is associated with increased risk of intensive care need and worse short-term prognosis [23].

Ocular involvement in aHUS is rare, found in approx. 4% of cases, but can be a severe complication when present. Unlike central nervous system manifestations, which occur in 8–48% of cases, ocular involvement has been documented only in isolated case reports [10,18,60,65]. Acute ophthalmic symptoms include reduced visual acuity, scotomas, ocular pain, diplopia and blurred vision, often with a sudden onset that may progress to partial or complete vision loss [73,74,75]. While some patients experience full visual recovery following treatment initiation, others may suffer from persistent visual deficits despite therapy [73,74,75].

### 3.4. Gastrointestinal Involvement

Digestive system complications are frequently observed in aHUS, with diarrhea occurring in approximately 50% of cases [61,76]. Individuals may present with nausea and vomiting, pancreatitis, gallstone formation, transaminitis, hepatitis, gastrointestinal bleeding, abdominal discomfort, eating difficulties, intestinal perforation and impaired bile flow [61,65]. In aHUS associated with anti-factor H antibodies, symptoms are present in over 80% of affected individuals, commonly including abdominal discomfort and nausea with vomiting [76,77]. Severe cases may involve pancreatic necrosis, ischemic colitis or terminal ileum perforation [78]. Gastrointestinal manifestations may precede other signs of TMA and should prompt early evaluation in patients with known complement dysregulation [79].

## 4. aHUS Diagnosis Criteria

aHUS is suspected when the TMA triad is present, consisting of MAHA, thrombocytopenia and organ damage [80]. While aHUS can occur at any age, regardless of whether it is inherited or acquired, other conditions can mimic TMA, including prosthetic heart valves, cardiopulmonary bypass, sickle cell crisis and metastatic emboli [81]. Early recognition is critical, as delays in diagnosis can lead to irreversible organ injury. The clinical overlap with other thrombotic microangiopathies necessitates systematic exclusion of alternative causes, particularly TTP and HUS [2].

In 2017, KDIGO classified TMA into primary and secondary types [57]. Primary TMA includes conditions with a well-established pathophysiology and treatment, such as TTP, caused by ADAMTS13 deficiency and aHUS [6]. Secondary TMA occurs due to underlying systemic conditions and often resolves when the primary cause is treated or removed [82]. Common secondary causes include Shiga toxin-producing *E. coli*, infections, pregnancy, transplantations, malignancies, autoimmune diseases, drugs and malignant hypertension [82,83]. These secondary forms are more frequent than primary TMA, with studies showing that 94% of cases are linked to conditions such as pregnancy (35%), infections (33%) and drug-related causes (26%) [82]. Since aHUS is a diagnosis of exclusion, it is only confirmed after ruling out TTP, HUS and other secondary TMA conditions [81,83]. The differential diagnostic criteria and treatment options distinguishing aHUS, TTP and HUS are presented in the Table 2 [84,85,86,87,88,89,90].

Specific laboratory thresholds guide this differential diagnosis: ADAMTS13 activity ≤ 10% confirms TTP, Shiga toxin detection confirms HUS and aHUS is considered when ADAMTS13 activity is >10%, Shiga toxin is negative and other causes are excluded [2].

In clinical practice, an algorithmic approach is recommended, which includes the following:

o Confirming the TMA triad;
o Testing ADAMTS13 activity;
o Evaluating for Shiga toxin-producing organisms (via stool PCR or serology);
o Assessing secondary causes (e.g., pregnancy, autoimmune disease, malignancy);
o If ADAMTS13 is >10%, Shiga toxin negative and no secondary triggers are found, aHUS is likely [10,12].

**Figure 1:**
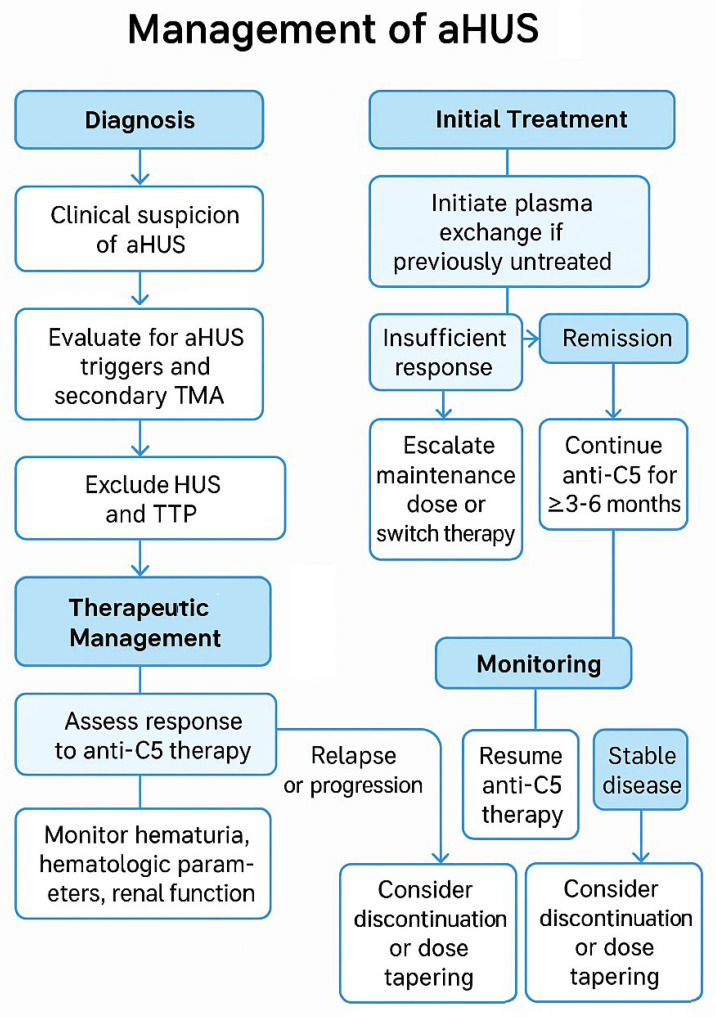
Diagnostic evaluation algorithm for aHUS.

A diagnostic approach is summarized in the flowchart presented in Figure 1.

At the time of clinical assessment, the etiology remains undetermined in most cases, necessitating a thorough differential diagnosis. Physicians must consider multiple potential causes and initiate targeted laboratory investigations to establish the underlying pathology and guide long-term management [12]. Since aHUS remains a diagnosis of exclusion, lacking a definitive biomarker or specific diagnostic test, its identification relies on systematically ruling out alternative thrombotic microangiopathies and secondary HUS causes [12].

Emerging biomarkers, such as soluble C5b-9, factor Ba and CH50, are under investigation to improve early diagnosis [91]. Elevated soluble C5b-9, Ba and CH50 reflect activation of the terminal, alternative and classical complement pathways, respectively, though none are yet standardized for routine clinical use [91,92].

The symptomatic profile of aHUS closely resembles other forms of TMA, as it arises due to red blood cell depletion and acute renal dysfunction [18,93]. This leads to manifestations such as pallor, exhaustion, growth impairment, swelling and lethargy. High blood pressure, whether newly emerging or an exacerbation of previously regulated hypertension, serves as a crucial diagnostic indicator that warrants careful attention [94].

aHUS predominantly impacts the kidneys, causing elevated creatinine, reduced eGFR, hypertension, proteinuria and hematuria. Renal impairment is a major laboratory feature, often marked by high creatinine, hematuria and proteinuria, occasionally reaching nephrotic levels [32,72]. Beyond renal involvement, additional systemic complications can arise, encompassing cardiovascular incidents, seizures, diffuse or localized neurological abnormalities, abdominal discomfort and nausea [67,93,94]. Approximately 20% of individuals with aHUS present with these extrarenal features. Coinciding infections are relatively frequent and may further complicate the clinical picture [27,94].

Diagnostic test results typically align with signs of intravascular red blood cell breakdown, including anemia, increased reticulocyte levels, diminished haptoglobin, elevated lactate dehydrogenase, hemoglobin in urine and a negative Coombs test (except in pneumococcal HUS) [93]. Furthermore, evidence of microvascular damage is observed, characterized by reduced platelet levels and red blood cell destruction with fragmented erythrocytes visible in peripheral blood smears [93]. A schistocyte count above 1% in peripheral blood smear strongly supports active TMA and should prompt immediate evaluation for aHUS or related disorders [95]. The first episode of aHUS can manifest at any stage of life and affects individuals of all sexes [25].

## 5. Management Strategy

Therapeutic strategies focus on halting complement-mediated endothelial injury, restoring organ function and preventing recurrence. Plasma therapy, including exchange and infusion, is a first-line treatment in aHUS, but its efficacy varies based on genetic factors [96,97]. While it may provide temporary hematologic remission, particularly in cases involving mutations in circulating complement regulators, it has not shown a definitive impact on long-term outcomes [27]. Many patients eventually progress to end-stage renal disease or mortality despite treatment [96]. Although plasma therapy can enhance hematologic parameters, it fails to inhibit the underlying complement overactivation, as indicated by consistently elevated markers of complement activity, inflammation, kidney damage and endothelial damage [98].

Eculizumab, a monoclonal anti-C5 antibody, inhibits C5 cleavage, preventing C5a and C5b-9 formation and blocking complement-driven inflammation and thrombosis [40]. Initially approved for paroxysmal nocturnal hemoglobinuria, its effectiveness in aHUS was first observed in an infant unresponsive to plasma therapy [99]. Eculizumab halted complement-mediated microangiopathy, leading to platelet count normalization and reduced lactate dehydrogenase levels [40,99]. Both adult and pediatric studies indicate that the early initiation of eculizumab significantly improves renal recovery in aHUS. These findings support current recommendations to administer eculizumab promptly after excluding other causes of TMA [24,100].

Prior to initiation, patients must be vaccinated against encapsulated organisms, including *Neisseria meningitidis*, *Streptococcus pneumoniae* and *Haemophilus influenzae* type B, ideally at least two weeks before the first dose [101,102]. If urgent treatment is required, vaccination should be administered concurrently with prophylactic antibiotics [102].

During treatment with C5 inhibitors, patients should undergo regular monitoring, including platelet count, serum creatinine, hemoglobin, lactate dehydrogenase and urinalysis [103]. In select cases, complement biomarkers, such as soluble C5b-9 or Ba may offer insight into ongoing complement activity, although these are not routinely used in clinical practice [91,92]. Monitoring during C5 inhibitor therapy includes regular assessments of platelet count, LDH, creatinine and hemoglobin [104]. Complement activity markers may provide additional information, although not routinely available [104].

In a systematic review, pregnancy-related aHUS has been identified as a major threat to both maternal and fetal well-being, frequently resulting in severe kidney impairment, the need for dialysis and critical complications, such as preeclampsia and HELLP syndrome, which can be life-threatening [105]. Fetal outcomes were also adversely affected, with many cases resulting in intrauterine growth restrictions and preterm births [105]. However, treatment with eculizumab demonstrated a substantial protective effect, significantly reducing the progression to chronic and end-stage kidney disease, thereby improving both maternal and fetal outcomes [105].

Ravulizumab, the first extended-duration C5 complement inhibitor, allows for an extended dosing interval of eight weeks, compared to the biweekly regimen required for eculizumab [106]. Clinical trials have demonstrated its non-inferiority in treating paroxysmal nocturnal hemoglobinuria and aHUS, with its extended half-life improving patient quality of life by minimizing the need for frequent intravenous infusions [106]. The restoration of platelet levels, serum lactate dehydrogenase and hemoglobin concentrations attained during the 26-week preliminary evaluation remained consistent through the latest follow-up, alongside persistent advancements in the estimated glomerular filtration rate (eGFR) and overall patient quality of life [107,108].

Across three studies, ravulizumab demonstrated improvements in blood clotting markers and kidney function in both pediatric and adult patients [107,109,110,111]. Its efficacy was comparable to eculizumab in children who transitioned between treatments, while adverse effects remained manageable, reinforcing its role as a reliable alternative for aHUS management [111].

In another study, pediatric aHUS patients transitioned from eculizumab to ravulizumab without relapse, following complications related to central vascular catheters [112]. One successfully underwent kidney transplantation with ravulizumab for complement inhibition [112]. The reduced infusion frequency allowed all patients to switch to peripheral access, minimizing the risks associated with long-term central vascular access while maintaining disease control for 2–4 years [112].

An analysis of aHUS in pregnancy highlights the importance of early detection and appropriate management to prevent severe complications, such as renal failure. Given the overlap with other thrombotic microangiopathies, timely laboratory testing is essential, though plasma exchange is often used initially due to diagnostic uncertainty. However, evidence suggests that eculizumab remains the most effective treatment for complement-mediated TMA [113].

Following the resolution of the pathology in patients receiving complement inhibitors, the question of whether to continue treatment indefinitely or discontinue it inevitably arises. The risk of aHUS recurrence after stopping eculizumab is estimated at 20–30%, with relapse being more frequent in individuals carrying pathogenic variants in complement-related genes compared to those without genetic mutations [114,115,116]. Currently, no established guidelines determine which patients are suitable candidates for therapy discontinuation [115].

Several studies have suggested that a genetics-informed approach may help guide safe discontinuation decisions, particularly in patients without high-risk mutations and with sustained remission [114,117,118]. Monitoring should be intensified in the first 6 months after withdrawal [119]. If treatment is halted, vigilant monitoring is essential to detect early signs of TMA recurrence and prevent progressive organ damage [114,115,116].

Alternative or adjunctive therapies, including corticosteroids, low-molecular-weight heparin and fresh frozen plasma, may be considered in specific scenarios, such as overlapping autoimmune disease or when eculizumab is unavailable, although evidence for their efficacy in aHUS remains limited [56].

**Figure 2:**
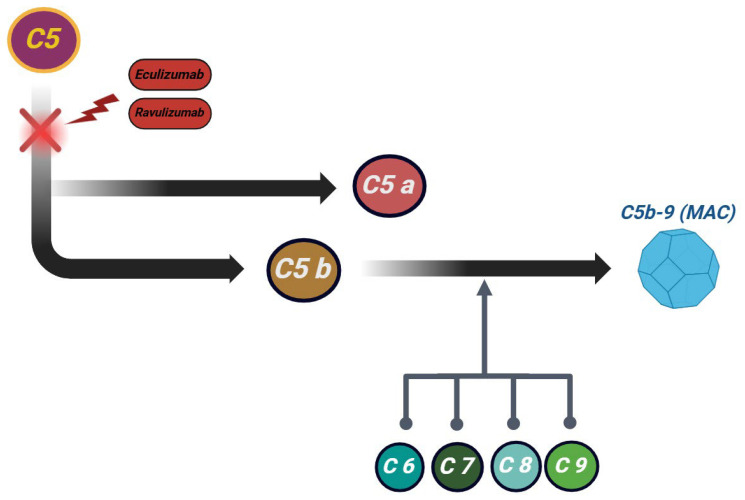
Molecular loci of principal complement cascade inhibitors.

Table 3 and Figure 2 provide the latest complement inhibitors, along with their most recent mechanisms of action [31,93,106,120].

## 6. Discussions

Globally, aHUS treatment protocols are evolving based on emerging clinical data, enabling a more informed assessment of the risks and benefits of therapy discontinuation [117]. Clinical decision analysis serves as a valuable tool for applying evidence-based approaches, helping clinicians make more objective and informed decisions regarding treatment termination [117].

In a clinical study, a 13-year-old boy presented with aHUS triggered by a gain-of-function mutation in C3 after a mild COVID-19 infection. Management included plasma exchange, but anti-C5 treatment was not necessary. This case adds to over 20 reported instances of COVID-19-associated aHUS, supporting evidence that SARS-CoV-2 proteins can activate the lectin and alternative complement pathways, potentially triggering disease even in mild infections [121]. Another case report on TMA presents a 23-year-old male with severe renal and cardiovascular complications due to an MCP/CD46 mutation [122]. Treatment with eculizumab, plasmapheresis and hemodialysis stabilized his condition [122]. The case underscores the importance of genetic testing in TMA management and highlights the need for further research to optimize targeted treatment strategies [122].

Pregnancy can trigger aHUS and complement-mediated TMA, complicating diagnosis due to symptom overlap with other thrombotic microangiopathies, being highlighted by two cases: one successfully managed with eculizumab after a CFI variant diagnosis and another with persistent complement activation postpartum despite negative genetic testing [123]. The findings also emphasize the importance of complement testing, genetic analysis and targeted therapy in diagnosing and managing pregnancy-associated aHUS and complement-mediated TMA [123].

Recent studies have significantly advanced our understanding of aHUS, particularly concerning its pathophysiology and management strategies. An international consensus emphasizes the pivotal involvement of complement system dysfunction in aHUS, advocating for the use of complement inhibitors as a frontline treatment [124,125]. This approach has demonstrated efficacy in halting disease progression and improving patient outcomes. However, important clinical gaps remain, including the lack of standardized criteria for initiating and discontinuing treatment, limited accessibility to genetic and complement testing in some settings and insufficient long-term data on newer therapies, such as ravulizumab [126].

Additionally, the high cost of lifelong therapy with complement inhibitors poses challenges for healthcare systems, particularly in low-resource countries. Although ravulizumab has improved patient convenience by reducing infusion frequency, its financial burden remains substantial [127]. Comparative health–economic analyses are urgently needed to assess the cost-effectiveness of extended-interval therapies versus standard regimens.

A study using FAERS data identified eculizumab-related adverse events, including expected effects, like fatigue and infections, as well as unexpected ones, such as aplastic anemia and kidney fibrosis [128]. The analysis revealed delayed onset in many cases and sex-based differences in some adverse reactions, emphasizing the need for ongoing monitoring and risk assessment in clinical practice [128].

A report on eculizumab discontinuation in aHUS patients found that 23% relapsed, with female gender, rare complement gene variants (MCP, CFH, CFI) and high soluble C5b-9 levels increasing risk [119]. Most patients recovered after re-initiation of treatment, although two experienced worsening chronic kidney disease. It has been concluded that a genetics-informed discontinuation strategy is feasible and safe in selected patients, improving their quality of life and reducing treatment costs [119].

In a real-world survey investigating treatment preferences and quality of life among aHUS patients, 94% of adult patients and all caregivers of pediatric patients reported a preference for ravulizumab over eculizumab [129]. This preference was primarily driven by ravulizumab’s reduced infusion frequency, which led to fewer disruptions to work, school and daily life and was associated with improved overall well-being [129].

Building on these findings, in an analysis of the Global aHUS Registry, patients who transitioned from eculizumab to ravulizumab maintained stable renal and hematologic parameters and experienced no additional occurrences of dialysis dependence, kidney transplantation or thrombotic microangiopathy [130]. Among the 49 patients assessed, 22% exhibited pathogenic mutations in complement-related genes, most commonly in CFH, while ravulizumab remained well tolerated, with minimal treatment-related adverse events and no cases of meningococcal infection or mortality [130].

Strengthening this perspective, another clinical trial evaluated the real-world safety and efficacy of switching adult aHUS patients from eculizumab to ravulizumab, analyzing 32 cases, including kidney transplant recipients [102]. Over a 12-month follow-up, no new thrombotic microangiopathy events or renal function deterioration were observed, while patients maintained hematologic stability [102].

## 7. Conclusions

As the understanding of aHUS continues to evolve, the integration of advanced molecular and genetic research remains critical for refining diagnostic accuracy and optimizing treatment approaches. While complement inhibition has revolutionized disease management, important clinical and operational challenges persist, particularly related to long-term therapy decisions and health system burden.

Genetic profiling is increasingly essential not only for diagnosis but also for risk stratification and therapeutic guidance, especially when considering treatment discontinuation. Emerging evidence supports the use of genetics-informed algorithms to determine which patients may safely withdraw from complement inhibitors, reducing exposure to costly and burdensome therapies.

Although therapies such as ravulizumab have improved quality of life by reducing infusion frequency, their financial impact remains substantial, especially in resource-constrained settings. This underscores the need for cost-effectiveness analyses and global access strategies to ensure equitable treatment. In parallel, novel therapeutic strategies, such as C3 inhibitors, RNAi therapies and agents targeting upstream complement components, are showing promise in clinical trials. These may offer more targeted, less immunosuppressive and potentially more affordable alternatives, though real-world data remain limited.

Looking ahead, continued investment in large-scale clinical trials and real-world data collection will be vital to refine evidence-based guidelines for aHUS management. A shift toward precision medicine, integrating clinical, biochemical and genetic data, will be key to improving patient outcomes while minimizing unnecessary treatment exposure. Strengthening collaborative research efforts and fostering innovation in diagnostics and therapeutics will be crucial in addressing the unmet needs in this rare but severe disease.

## 8. Future Directions

The future of aHUS management will increasingly rely on the integration of genetic, immunologic and clinical data to support early diagnosis, individualized treatment decisions and long-term follow-up. Advances in high-throughput sequencing technologies and artificial intelligence are expected to enhance our ability to identify high-risk individuals and predict therapeutic response more accurately.

Emerging therapeutic options targeting upstream components of the complement cascade, such as factor D, factor B or C3, hold promise for improved safety and efficacy. Oral agents currently in late-phase trials may also improve treatment accessibility and adherence by removing the need for intravenous infusions. These next-generation inhibitors may ultimately expand the treatment landscape, particularly for patients who are poor responders to terminal pathway blockade.

There remains a pressing need to establish standardized guidelines for treatment discontinuation, taking into account genetic risk factors, complement activity markers and the patient’s clinical trajectory. Biomarkers, such as soluble C5b-9, Ba or factor H–autoantibody complexes, may contribute to personalized monitoring strategies, although further validation is required before routine use.

Global registries and real-world observational cohorts will be instrumental in capturing long-term data on treatment durability, relapse risk, pregnancy outcomes and post-transplant recurrence. In parallel, strategies to improve treatment affordability, including biosimilars, cost-effectiveness modeling and equitable access programs, will be essential for the global implementation of complement inhibitor therapies.

Expanding awareness of aHUS among clinicians outside of nephrology and developing streamlined diagnostic pathways will be key to reducing diagnostic delays and improving outcomes, especially in acute and perinatal care settings.

## Figures and Tables

**Table 1:** Key complement gene mutations and their clinical implications in aHUS.

Gene	Protein Affected	Mutation Type	Estimated Frequency in aHUS	Penetrance	Clinical Implications	Source
**CFH**	Complement factor H	Loss-of-function	~20–45%	~50%	Early onset; poor prognosis; high recurrence post-transplant	[14,38,39]
**MCP**	Membrane cofactor protein	Loss-of-function	~10%	~20%	Often triggers with infection; better prognosis; low recurrence after transplantation	[14,38,40]
**CFI**	Complement factor I	Loss-of-function	~5–10%	Variable	Intermediate severity; incomplete penetrance; may coexist with other variants	[14,25]
**C3**	Complement C3	Gain-of-function	~4–10%	Moderate	Severe presentation; poor prognosis; more resistant to plasma therapy	[14,41]
**CFB**	Complement factor B	Gain-of-function	<1%	Unknown	Rare; usually severe; limited data available	[14,40]
**THBD**	Thrombomodulin	Loss-of-function	~3–5%	Low	May present with mild phenotype; data on recurrence limited	[14,42]

**Table 2:** Comparative diagnostic features and treatment strategies for aHUS, TTP and HUS.

*Feature*	*aHUS*	*TTP*	*HUS*
Etiology	Inherited or acquired imbalance in the regulation of the alternative complement pathway	Severe deficiency of ADAMTS13 enzyme activity (≤10% of normal), often due to autoantibodies	Various triggers, including infections (e.g., Shiga toxin-producing *E. coli*), drugs or systemic diseases
Microangiopathic Hemolytic Anemia	Present; characterized by schistocytes on peripheral smear and elevated lactate dehydrogenase	Present; similar findings as in aHUS	Present; similar findings as in aHUS
Thrombocytopenia	Present; platelet count typically < 150,000/μL	Present; often severe with platelet count < 30,000/μL	Present; platelet count decreased but not as low as in TTP
Acute Kidney Injury	Common and often severe; elevated serum creatinine and proteinuria	Less common; renal involvement is usually mild	Prominent; often severe renal impairment
Neurological Symptoms	Can occur but are less frequent and less severe than in TTP	Common; may include confusion, seizures and focal deficits	Less common; when present, may include irritability and seizures
ADAMTS13 Activity	Typically > 10% of normal activity	Severely reduced (≤10% of normal activity)	Normal
Shiga Toxin Detection	Negative	Negative	May be positive if associated with *E. coli* infection
Complement Level	Often decreased (e.g., low C3 and C4)	Normal	Typically normal
Family History	May have a family history of similar episodes	Usually absent	Usually absent
Treatment Approach	Eculizumab or ravulizumab (C5 inhibitors); supportive care; vaccination prior to therapy; consider genetic testing; dialysis if needed	Urgent plasma exchange; corticosteroids; rituximab or caplacizumab in selected cases	Supportive care; avoid antibiotics and antimotility agents; dialysis if needed

**Table 3:** Complement inhibitors used in aHUS: targets, mechanisms and clinical applications.

*Target in Complement Pathway*	*Level of Action*	*Examples of Complement Blockers*	*Mechanism of Action*	*Administration and Indication*
C3	Early stage of complement activation (alternative pathway)	Pegcetacoplan, APL-2	Inhibits C3 cleavage, preventing complement cascade activation and C3 convertase formation.	Subcutaneous; in trial phase for complement diseases
C5	Late stage of complement activation	Eculizumab, ravulizumab, crovalimab	Blocks C5 cleavage, preventing C5a (pro-inflammatory) and C5b, which leads to MAC formation. Crovalimab is an anti-C5 monoclonal antibody with subcutaneous administration.	IV (biweekly or every 8 weeks); approved in aHUS
Membrane Attack Complex (MAC—C5b-9)	Final stage of complement cascade	Nomacopan, zilucoplan (under investigation)	Nomacopan inhibits both C5 and leukotriene B4 (LTB4), reducing inflammation and MAC formation. Zilucoplan is a C5 inhibitor.	Under investigation
Factor D	Key regulator in the alternative pathway	Danicopan (ACH-4471)	Inhibits factor D, blocking C3 convertase activation and stopping the complement cascade.	Oral; in trial phase
Factor B	Part of the alternative pathway C3 convertase	Iptacopan (LNP023)	Inhibits factor B, preventing further complement activation.	Oral; ongoing trials
Hepatic C5 Synthesis	RNA interference-based inhibition	Cemdisiran	Reduces C5 production in the liver via RNA interference (siRNA), lowering circulating C5 levels and decreasing complement activation.	Subcutaneous; in development

## Data Availability

Not applicable.
